# Advisory Committee on Immunization Practices Recommended Immunization Schedule for Adults Aged 19 Years or Older — United States, 2015

**Published:** 2015-02-06

**Authors:** David K. Kim, Carolyn B. Bridges, Kathleen H. Harriman, Advisory Committee on Immunization Practices (ACIP), ACIP Adult Immunization Work Group

**Affiliations:** 1 Immunization Services Division, National Center for Immunization and Respiratory Diseases, CDC; 2 California Department of Public Health

In October 2014, the Advisory Committee on Immunization Practices (ACIP) approved the *Recommended Immunization Schedule for Adults Aged 19 Years or Older, United States, 2015*. This schedule provides a summary of ACIP recommendations for the use of vaccines routinely recommended for adults aged 19 years or older in two figures, footnotes for each vaccine, and a table that describes primary contraindications and precautions for commonly used vaccines for adults. Changes in the 2015 adult immunization schedule from the 2014 schedule included the August 2014 recommendation for routine administration of the 13-valent pneumococcal conjugate vaccine (PCV13) in series with the 23-valent pneumococcal polysaccharide vaccine (PPSV23) for all adults aged 65 years or older (1), the August 2014 revision on contraindications and precautions for the live attenuated influenza vaccine (LAIV) (2), and the October 2014 approval by the Food and Drug Administration to expand the approved age for use of recombinant influenza vaccine (RIV) (3). These revisions were also reviewed and approved by the American College of Physicians, American Academy of Family Physicians, American College of Obstetricians and Gynecologists, and American College of Nurse-Midwives.

*Recommendations for routine use of vaccines in children, adolescents, and adults are developed by the Advisory Committee on Immunization Practices (ACIP). ACIP is chartered as a federal advisory committee to provide expert external advice and guidance to the Director of the Centers for Disease Control and Prevention (CDC) on use of vaccines and related agents for the control of vaccine-preventable diseases in the civilian population of the United States. Recommendations for routine use of vaccines in children and adolescents are harmonized to the greatest extent possible with recommendations made by the American Academy of Pediatrics (AAP), the American Academy of Family Physicians (AAFP), and the American College of Obstetricians and Gynecologists (ACOG). Recommendations for routine use of vaccines in adults are harmonized with recommendations of AAFP, ACOG, and the American College of Physicians (ACP). ACIP recommendations adopted by the CDC Director become agency guidelines on the date published in the Morbidity and Mortality Weekly Report (MMWR). Additional information regarding ACIP is available at http://www.cdc.gov/vaccines/acip.*

The 2015 adult immunization schedule contains the following changes from the 2014 schedule:

- Figure 1, the recommended adult immunization schedule by vaccine and age group, has been revised to designate PCV13 for adults aged 65 years or older as “recommended” (from the previous “recommended if some other risk is present”). Figure 2, showing vaccines that might be indicated for adults on the basis of medical and other indications, is unchanged.
- The footnotes for pneumococcal vaccination have been revised to provide algorithmic, patient-based guidance for the health care provider to arrive at appropriate vaccination decisions for individual patients.
- The footnote for influenza vaccination has been updated to indicate that adults aged 18 years or older (changed from adults aged 18 through 49 years) can receive RIV. (The upper age limit for LAIV remains 49 years.) A list of currently available influenza vaccines is available at http://www.cdc.gov/flu/protect/vaccine/vaccines.htm.
- Table 1, showing contraindications and precautions to commonly used vaccines in adults, has been revised to update the section on LAIV to reflect the changes in the ACIP recommendations for the 2014–15 influenza season. These changes include moving “influenza antiviral use within the last 48 hours” from the precautions column to the contraindications column, and moving asthma and chronic lung diseases; cardiovascular, renal, and hepatic diseases; and diabetes and other conditions from the contraindications column to the precautions column. Immune suppression, egg allergy, and pregnancy remain contraindications for LAIV.

Details on these updates and information on other vaccines recommended for adults are available under *Adult Immunization Schedule, United States, 2015* at http://www.cdc.gov/vaccines/schedules and in the *Annals of Internal Medicine* (4). The full ACIP recommendations for each vaccine are not included in the schedule because of space limitations but are available at http://www.cdc.gov/vaccines/hcp/acip-recs/index.html.
